# Signaling Strategies of Malaria Parasite for Its Survival, Proliferation, and Infection during Erythrocytic Stage

**DOI:** 10.3389/fimmu.2017.00349

**Published:** 2017-03-28

**Authors:** Rani Soni, Drista Sharma, Praveen Rai, Bhaskar Sharma, Tarun K. Bhatt

**Affiliations:** ^1^Department of Biotechnology, School of Life sciences, Central University of Rajasthan, Ajmer, India

**Keywords:** malaria, *Plasmodium*, cyclic nucleotide signaling, toll-like receptor, calcium signaling, glycosylphoshatidylinositol, cytoadhesion

## Abstract

Irrespective of various efforts, malaria persist the most debilitating effect in terms of morbidity and mortality. Moreover, the existing drugs are also vulnerable to the emergence of drug resistance. To explore the potential targets for designing the most effective antimalarial therapies, it is required to focus on the facts of biochemical mechanism underlying the process of parasite survival and disease pathogenesis. This review is intended to bring out the existing knowledge about the functions and components of the major signaling pathways such as kinase signaling, calcium signaling, and cyclic nucleotide-based signaling, serving the various aspects of the parasitic asexual stage and highlighted the Toll-like receptors, glycosylphosphatidylinositol-mediated signaling, and molecular events in cytoadhesion, which elicit the host immune response. This discussion will facilitate a look over essential components for parasite survival and disease progression to be implemented in discovery of novel antimalarial drugs and vaccines.

## Introduction

*Plasmodium falciparum* is one of the major afflictions to the human health. The annual report of malaria speculated around 2.1 million cases of disease and more than 0.4 million mortality cases in 2015 (1). The emergence of drug resistance species appended the severity of the problem. The intracellular inhabitation of *Plasmodium* makes the substantial modification in the host cell environment. After exoerythrocytic schizogony, the merozoites are released from hepatocytes into the blood stream and targeted to host erythrocytes, which marks the beginning of erythrocytic phase. Inside the erythrocyte, they multiply asexually and released following the rupture of RBC. These newly released merozoites make the recurrence of the same process for the fresh erythrocytes (2). Infection of fresh erythrocyte requires the egress from infected erythrocyte and reinvasion to fresh one. During egress and internalization process, multiple molecular interactions between the surface proteins of merozoites and receptors on the host erythrocytes come into play (3). Moreover, the parasitic entry into the host cell modulates the host environment to suit its own needs and to stay clear from the host defense. The modulation processes are coupled with a well-defined signaling mechanism, which can be described at cellular and molecular levels (4, 5). A few of the large repertoire of parasite proteins involved in modulating the host signaling pathways are summarized in the Table 1. Despite of unraveling of the functions and involvement of molecules in signaling pathways during the parasite life cycle, certain proteins remained uncharacterized. The analysis of different signaling mechanism during the asexual erythrocytic stage (6, 7) will be advantageous in understanding the strategies used by parasite to thrive successfully in the host, which would give novel input in planning an effective antimalarial therapeutic approach.

**Table 1:** ***Plasmodium* protein triggering signals associated with modulation in host response**.

*Plasmodium* protein	Associated signaling pathway of host	Response	Reference
*Pf*B190 (*Pf*SEL1) and PF14_0462 *Pf*N462 (*Pf*SEL2)	Notch signaling pathway	T cell differentiation to Th1 cell leads to weak antibody response	Singh et al. (8)
Epoxide hydrolase 1 (*Pf*EH1) and 2 (*Pf*EH2)	Epoxide signaling of RBCs	Perturbed vascular signaling and inflammation	Spillman et al. (9)
*Pf*EMP1	MAP kinase dependent on Src family kinase	Modulation of cytoadherence property in endothelium	Ho and White (10); Yipp et al. (11)
Pf-IRBC/*Pf*EMP1 and other unknown membrane associated proteins	Nuclear factor kappa-light-chain-enhancer of activated B cells signaling pathway	Increase intercellular adhesion molecule 1 expression on brain endothelium increase sequestration Pf-IRBC	Tripathi et al. (12)
Unknown	P-21 activated protein kinase-MEK signaling pathway	Activation of the “novel permeation pathway” required for nutrient uptake in to the infected RBC	Sicard et al. (13)
*Pf*EMP1 subtypes containing domain cassettes 8 and 13	Activated protein C signaling	Interfere with the activation of cytoprotective and anti-inflammatory pathways	Turner et al. (14)
Unknown	Toll-like receptor (TLR) signaling through TLR9	Activation of regulatory T cells contribute to immune evasion	Hisaeda et al. (15)
Unknown	TLR signaling through TLR7	Increased production of pro-inflammatory cytokine interferon (IFN)-1, interleukin (IL)-12, and IFN-γ	Baccarella et al. (16)
Tyrosyl-tRNA synthetase (*Pf*TyrRS)	–	Enhanced secretion of the pro-inflammatory cytokines tumor necrosis factor-α and IL-6	Bhatt et al. (17)

## Cyclic Nucleotide-Based Signaling During Malaria

Signals from the extracellular environment are transmitted inside the cell through the secondary messenger molecules like cyclic adenylyl monophosphate (cAMP) and cyclic guanylyl monophosphate (cGMP). The homologous genes for enzymatic components involved in cyclic nucleotide-based signaling like adenylyl cyclase (AC), guanylyl cyclase (GC), cGMP-dependent protein kinase [protein kinase G (PKG)], and a regulatory and catalytic subunit of cAMP-dependent protein kinase, and nucleotide phosphodiesterase (PDE) have been identified (18, 19) in malaria parasite.

### *Plasmodium falciparum* Protein Kinase A (*Pf*PKA) and cAMP

The first evidence of the cAMP signaling in malaria parasite arose through an experimental study in which the addition of external cAMP to *Plasmodium* culture was shown to positively affect the exflagellation or gametocyte formation during the ring stage of the parasite (20). However, the parasitic AC differs biochemically from that of host counterpart. Forskolin and Alf4, the activators of mammalian AC, and GTPγs, the activator of G protein, are unable to cause stimulation in parasitic AC (21). Moreover, expression of G stimulatory α have been demonstrated in early asexual stage and mature sexual stage. So, it was assumed that G protein might be implicated in the signaling during gametogenesis. However, this finding leads to a dilemma because the *Plasmodium* genome is devoid of gene corresponding to the G protein (22).

**Figure 1:**
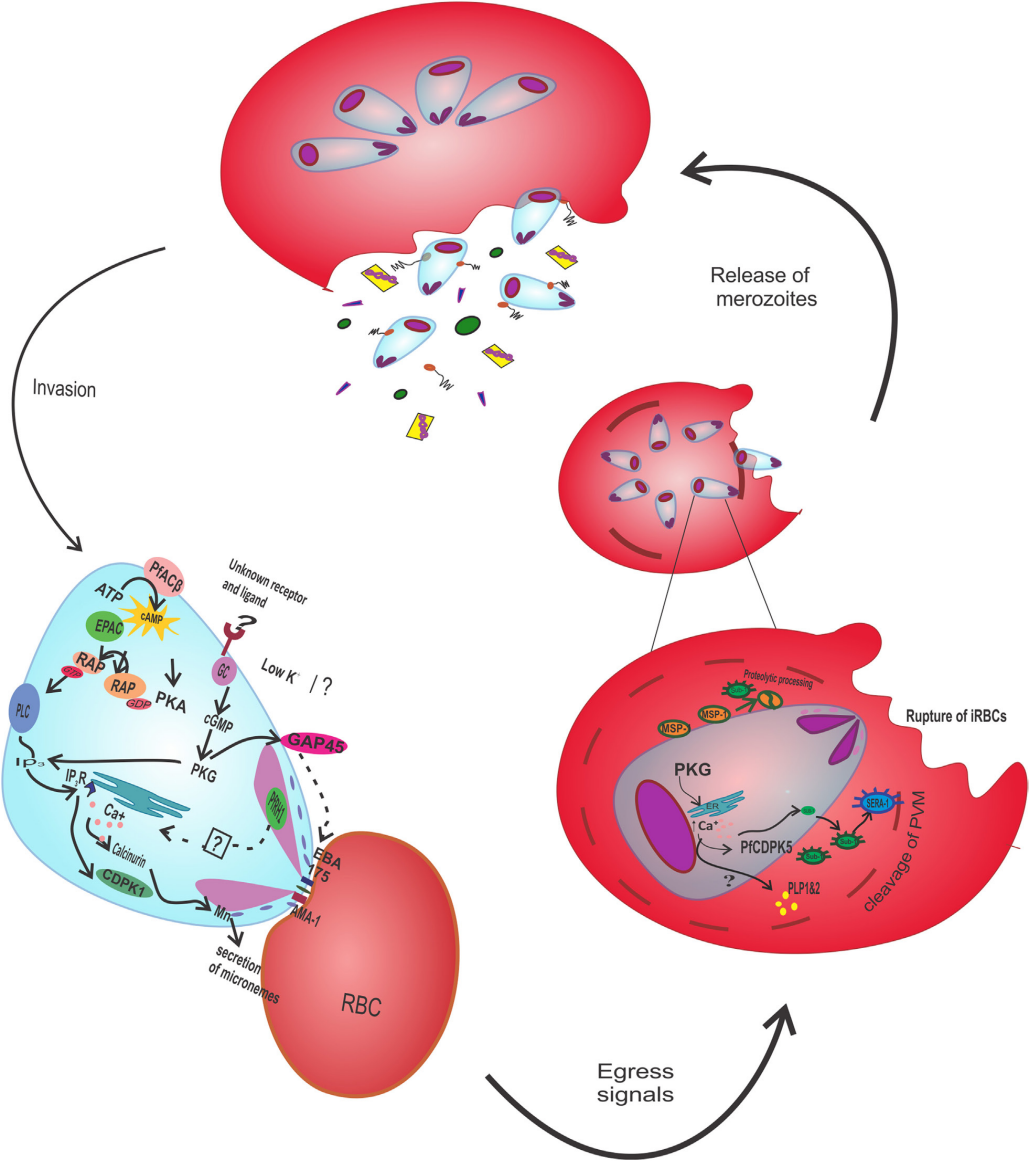
**Signaling during egress and invasion of merozoites: invasion is mediated through the secretion of apical organelle, microneme and rhoptry organelle containing AMA-1 and GAP45, and EBA175**. *Pf*ACβ triggers the cAMP level, boosting Epac pathway thereby phosphorylating RAP-GDP into RAP-GTP. RAP-GTP activates PLC to produce IP3 that binds to IP3R on endoplasmic reticulum, releasing calcium ions. Calcium ions bind to calcineurin and also activate CDPK1, required for the discharge of microneme and rhoptry. During egress, the cleavage of parasitophorous vacuole and rupture of infected erythrocyte require the proteolytic processing of *Pf*SERA and *Pf*MSP-1. The activation of *Pf*SERA and *Pf*MSP-1 occurs through the proteolytic activity of *Pf*SUB-1, caused by increment of intracellular calcium ions in response to PKG. Calcium ions also regulates the release of PLP1 and PLP2 and activates *Pf*CDPK5. AMA-1, apical membrane antigen 1; GAP45, glideosome-associated protein 45; EBA175, erythrocyte-binding antigen 175; *Pf*ACβ, *Plasmodium falciparum* adenylyl cyclase-β; cAMP, cyclic adenylyl monophosphate; Epac, exchange protein activated by cAMP; Rap-GDP, Ras proximate guanylyl diphosphate; PLC, phospholipase C; IP3, inositol 1,4,5-trisphosphate; IP3R, IP3 receptor; CDPK1, calcium dependent protein kinase 1; *Pf*SERA, *Plasmodium falciparum* serine-like repeat antigen; *Pf*MSP-1, *Plasmodium falciparum* merozoite surface protein; *Pf*SUB-1, *Plasmodium falciparum* subtilisin like protease; PKG, protein kinase G; PLP, perforin-like protein.

Adenylyl cyclase and cAMP signaling were demonstrated to play an important role during the infection of hepatocytes by sporozoites. The migration of sporozoites across the host hepatocytes results in their activation and triggering of apical regulated exocytosis. The sporozoites can be activated externally by calcium ionophore, which are then able to infect liver cell without migration (23). The gene knockout experiment of ACα in *Plasmodium berghei* explained the prevention of the exocytosis along with the reduced infectivity. However, the results were reciprocated after the reintroduction of the ACα gene into the mutant. Thus, the involvement of cAMP-mediated signaling in the initial phase of infection was confirmed. Besides this, the ACα also share homology with K^+^ channels, which are required for exocytosis in sporozoites (24). Not only sporozoites but also merozoites invasion process also involves cAMP-dependent signaling. During invasion, there occurs the formation of tight junctions with host cells, which leads to the secretion of apical organelle containing several proteins like gliding-associated protein 45 and apical membrane antigen 1 (AMA-1) (25, 26). The whole event of invasion is regulated by the cAMP-dependent phosphorylation of protein AMA-1 mediated by *Pf*PKA (Figure 1). The mutational analysis of AMA-1 showed the hampering of the invasion process due to a change in phosphorylation site (serine 610) (27). It was evidenced from another study that merozoite proteins, particularly localized to the microneme and rhoptry organelle, get secreted and interact with receptors on fresh erythrocytes. The secretion of proteins involves a stepwise signaling cascade initiated due to the exposure of the low K^+^ extracellular environment (28–32). Low K^+^ triggers activation of *Pf*ACβ followed by an increase in cAMP. The cAMP activates Epac (exchange protein activated by cAMP) pathway, which subsequently cause an increase in *Pf*PKA coupled with elevation of Ca^2+^ level (33). In the Epac pathway, Ras-proximate-1 (Rap1) converted to Rap1-GTP and further activates phospholipase C (PLC). The activated PLC induces calcium-dependent protein kinase 1 and calcineurin, which eventually leads to the secretion of microneme and rhoptry proteins (33–35) (Figure 1). Intense analysis of the pathway and its regulating components rendered a better understanding of overall mechanism, which can be implemented for inhibiting parasitic growth, invasion, and malaria prevention.

The parasitic infection remodels the membrane of host erythrocytes for the nutrient acquisition and maintaining the balance of electrolytes. The electrophysiology-based studies indicated a peak of conductance of anionic channels during *Plasmodium* infection (36). The conductance of anions across the infected host cell membrane is mainly regulated by cAMP signaling. The experimental addition of PKA and ATP to the uninfected erythrocyte caused the upregulation of anion conductance (26), while the process overturned on the addition of alkaline phosphatase (ALP) or by dephosphorylation (37, 38). In this contest, the dependency of anion channel regulation on cAMP was further confirmed through an experiment with *Pf*PKI (H89 cAMP-dependent protein kinase inhibitor) or by developing transgenic parasite with higher expression of *Pf*PKA-R (phosphokinase A-regulatory subunit), which binds to cAMP, thereby downregulating the process and directly affecting the parasitic growth (39, 40). The signaling cascade either involves parasitic components or host is still controversial. Also, little is known about the actual substrates of PKA. The above discussion presents the essentiality of cAMP. Cyclic nucleotides are produced by AC and GC on hydrolysis, which requires PDE (19, 41). Therefore, PDE regulates the production and functioning of cAMP and cGMP. In *P. falciparum, Pf*PDE1 was the first-reported PDE, which is specific for cGMP (42). Among various PDE types, PDE4 is predominant in the immune cells. Implementation of PDE4 inhibitors was found to enhance chemokines production and elicit inflammatory response. To delineate the regulatory mechanism of cyclic nucleotide, parasite-specific PDE inhibitors were developed. Zaprinast, a PDE5 inhibitor, and 5-benzyl-3-isopropyl-1H-pyrazolo[4, 3-d] pyrimidin-7(6H)-one, inhibitor of *Pf*PDEα, were potentially most effective inhibitors blocking the parasite proliferation (43). PDE inhibitors can be explored in developing antimalarial therapy (44).

### *Pf*PKG and cGMP

The protein sequence analysis of *Pf*PKG with respect to vertebrate counterpart revealed certain differences between their sequence features. For instance, the presence of three cAMP/cGMP binding motif, a degenerated cGMP binding motif and lack of leucine zipper motif required for dimerization and insensitivity towards cGMP analogue (45, 46). Along with its role in gametogenesis, cGMP has been demonstrated to express its functionality during the ring and schizogony stage (45). A known PKG inhibitor, 4-[2-(4-fluorophenyl)-5-(1-methylepiperidine-4-yl)-1H pyrrol-3-yl] pyridine (compound 1), has a retarding effect on parasitemia level (47, 48). In contrast, the mutant strain with genetically modified *Pf*PKG showed normal development in the presence of inhibitor compound 1. These findings established the key role of cGMP signaling during the asexual phase of parasite development.

Conditional knockout study carried on *P. berghei* PKG (*Pf*PKG) at late liver sporozoites (LS) stage depicted that parasite can infect hepatic HePG2 cell lines, but failed to get released from hepatocytes as merosome. Sporozoites at this stage elicited protective immune response in the host (49). Thus, it reflects an essentiality of cGMP signaling during the formation and release of merosome. So far, the precise role of PKG and trigger for its stimulation is not clear. A deep insight into the signaling involved in LS would be opportune to control disease pathologies at the erythrocytic stage and can be advantageous in search of prospective medication of malaria and preerythrocytic vaccine development (49).

During the egress cascade, PKG is required in proteolytic processing of proteins such as proteolytic processing of *P. falciparum* serine-like repeat antigen (*Pf*SERAs) and *Pf*MSP-1 by *P. falciparum* subtilisin-like protease *(Pf*SUB1) (Figure 1), which in turn are involved in merozoite egress and secretion of apical organelle (50, 51). The proteolytic processing of *Pf*MSP-1 was prevented with the inhibitor of *Pf*PKG, compound 1 without affecting the activity of SUB1. Noticeable, in an experiment involving schizonts deficient in *Pf*CDPK5 showed normal processing of *Pf*MSP-1 in the presence of compound 1. Therefore, it indicates the role of *Pf*CDPK5 downstream to PKG (52). The phosphoproteomics and chemical genetic approach involving the use of PKG inhibitor, compound 2 4-[7-[(dimethylamino) methyl]-2-(4-fluorophenyl)imidazo[1,2-α]pyridin-3-yl]pyrimidin-2-amine along with strain containing PKG mutant allele (*Pf*PKG_T_618_Q_), resistant to compound 2 further demonstrated the role of PKG in the signaling event. Comparative analysis of the effect of treatment with compound 2 in wild-type and *Pf*PKG mutant brought out various cellular targets of *Pf*PKG involved in egress and invasion (53). The significance of *Pf*PKG upstream to calcium signaling was indicated by a study in which phosphorylation of *Pf*CDPK1 occurred in a PKG-dependent manner (50, 53). Moreover, PKG plays a significant role in regulating the cytosolic calcium signaling both during egress and at the asexual stage (54). The key importance of *Pf*PKG in phosphorylation of the substrates gives a reflection that both *Pf*PKG and its substrates are the therapeutic candidates in malaria treatment and in transmission blocking (55).

### ATP as Signaling Molecule in Infected Erythrocytes

During malaria, extensive modification in erythrocytes is accompanied by changes in membrane permeability (56, 57). It has been well documented that hypoxic signals or surface deformation of RBC accounts for the release of ATP (58, 59). An elevated level of ATP has been recorded both in the extracellular environment and in the cytoplasm of host and parasite (60, 61). Parasitemia is proved to be directly linked to level of ATP (62). Any diminution of ATP in the medium rendered the *Plasmodium* ineffective to infect the fresh erythrocytes (60, 63, 64). The essentiality of ATP in the invasion process of parasite was confirmed by thwarting the entry of the parasite through inhibitor-mediated blocking of purinergic receptor and the addition of apyrase (65). The underlying mechanism of this inhibition involved the boosting up of the cytoplasmic calcium levels by extracellular ATP (65). The effect of purinergic signaling is mediated through phosphorylation of skeleton protein like spectrin present in erythrocyte (64, 65). Purinoceptor signaling is linked to induction of the new permeation pathway. Therefore, purinoceptors antagonist, suramin, reduces the membrane permeability and leads to deterioration in parasitic growth, both *in vivo* and *in vitro* (66). Purinoceptor blocker, suramin, and pyridoxal phosphate-6-azophenyle-2′,4′-disulphonic acid alter the proteolytic processing of protein like MSP-1, which is involved in the invasion, while the addition of ATP was found to trigger the intracellular proteolytic event in *P. berghie* and *Plasmodium yoelii* (67). It suggests the involvement of purinergic receptors in ATP signaling. The purinergic receptors that are expressed on the parasite surface are phylogenetically distinct from human counterparts. Thus, these receptors can be exploited as targets so as to design inhibitors of parasite invasion process (68, 69). ATP is also thought to be involved in the host-induced inflammation following the malaria infection (70).

## P-21 Activated Protein Kinase (PAK)-MEK Signaling Pathway in Infected Erythrocytes

In eukaryotes, the regulation of cell cycle is dependent on mitogen-activated protein kinases (MAPKs). In this pathway, signals are transmitted successively to its respective components, MAPKKKs (MEKKs), MAPKKs [MEK/extracellular signal regulated kinase (ERK)], and finally to MAPK (71, 72). The *Plasmodium* kinome study revealed the presence of two homologs of human MAPK, namely, *Pf*map-1 (73, 74) and *Pf*map-2 (74, 75). *Pf*map-1 is expressed in both asexual and gametocyte stage, while the expression of *Pf*map-2 is found in gametocytes only. The reverse genetic-based approach demonstrated the essentiality of *Pf*map-2 in the asexual development of *P. falciparum* (74). The sequence alignment depicted that the homology of gene *Pf*PK7 with human MAPKK3 at its *C*-terminal region and the *N*-terminal region was aligned with fungi PKA. The assay conducted to analyze the inhibition *via* phosphokinase inhibitors, namely, PKI and H89 showed that *Pf*PK7 activity was not affected. Similarly, MEK inhibitor, U0126, had no inhibitory effect on *Pf*PK7 activity. It was suggested that *Pf*PK7 is not an ortholog of MAPKK due to the absence of MAPKK activation site (76). The data provide an evidence of the absence of a regular MAPK pathway in *Plasmodium*. Due to the absence in parasite, the role of MEK of host erythrocytes in parasite development was hypothesized and confirmed. The immunological experiment evidenced the modulation of the host erythrocyte MAPK pathway. When compared, the level of phosphorylated MEK in infected erythrocytes was found much higher than uninfected ones. Moreover, MEK inhibitors, U0126 and PD184352, had parasiticidal effect on trophozoite, while the invasion process remained ineffective (13). It was found that activation of MEK is relying on MEKK-independent mechanism, which involves PAK (77). The involvement of PAK-1 was evident by observing the inhibition of parasitic growth due to reduction in the phosphorylation of MEK-1 by the use PAK-1 inhibitor, IPA-3 (78). The activation of PAK-1 occurs as a consequence of erythrocyte remodeling during parasite infection (79, 80). Furthermore, the importance and requirement of MEK-1 and substrate of MEK-1-PAK-1 pathway for parasite are yet obscured (13). Targeting the human kinase would provide a discrete strategy for the development of antimalarial therapy. It could be advantageous as many kinase inhibitors are known to pass the phases of clinical trials as anticancerous agents. If such inhibitors have “cidal” effect on parasite, then they can be used as an antimalarial in a cost-effective manner. The overall lengthy process of drug development can also be reduced. Second, targeting host protein will aid in circumventing the problem of drug resistance (13). Most common examples are the pyridinyl imidazoles SB203580 and SB202190 known to inhibit the activation of human p38MAPK. These p38MAPK inhibitors were found to be inhibitory for protozoan parasite as well. Similarly MAPK p38 inhibitors like pyridinyl imidazole RWJ67657 and pyrrolobenzimidazole RWJ68198 impede the growth of *P. falciparum*.

## Ca^2+^ Signaling

Ca^2+^ is one of the important secondary messenger molecule involved in the signal transduction. It plays multiple roles in different aspects of parasite lifecycle, such as egress, invasion, growth, development, motility, and secretion (64, 81, 82). Therefore, maintenance of Ca^2+^ homeostasis is crucial for the parasite survival. Ca^2+^ ions regulate varied cellular events by binding to the effector molecules. But it is difficult to characterize the components of Ca^2+^-dependent signaling due to the lack of homology of effector molecules between *Plasmodium* and higher eukaryotes (83). Some effectors like Ca^2+^ transporters, *Pf*CHA (PF3D7_0603500), similar to Ca^2+^/H^+^ exchanger (84), *P. falciparum* sarco-endoplasmic reticulum calcium ATPase (SERCA-type ATPase), *Pf*ATP6 (PF3D7_0106300), and *Pf*ATP4 (85) have been identified, but their functions need to be investigated.

### Ca^2+^ Signaling during Egress

Live cell fluorescent video microscopy involving the use of fluo-4 Ca^2+^ probe, Ca^2+^ chelators, and inhibitors of Ca^2+^ ATPase revealed the differential role of various effector molecules in egress and the invasion of the merozoites. It has been shown that before egress of merozoites there is a constant increase in Ca^2+^ level in the cytoplasm of both parasite and infected RBC (iRBC) independent of extracellular Ca^2+^ ions.

Inhibition of Ca^2+^ ATPase from ER inhibitors and addition of Ca^2+^ ionophore enhanced the process of egress, while chelating agents like bis(o-aminophenoxy)ethane-*N*,*N*,*N*′,*N*′-tetra acetic acid inhibited the process of egress (64, 86). The mechanism of inhibition involved the prevention of permeabilization of host membrane. The Ca^2+^ is found to regulate the release of perforin-like protein 1 (PLP1) from microneme to the membrane where it begins its lytic activity. Likewise, the expression of PLP2 also occurs during the asexual blood stage, but its role is not clear. The reverse genetic studies would be helpful in disclosure of the details of PLP1 and PLP2(86). *P. falciparum* subtilisin-like protease (*Pf*SUB1) is another effector of calcium signaling, which is found to be required for the *Pf*SERAs. The inhibition of *Pf*SUB1 by the use of Ca^2+^ chelators prevented the rupture of the parasitophorous vacuole and consequently the egress of merozoites (86).

### Ca^2+^ Signaling during Invasion

The invasion process requires the discharge of rhoptry and microneme, which is dependent on the elevated levels of Ca^2+^. On exposure to low K^+^ level, cytosolic Ca^2+^ level increases through PLC and it triggers the export of erythrocyte-binding antigen 175 (EBA175) and AMA-1 from microneme to merozoite surface (Figure 1). The interaction with the receptors of RBC brings the increased level of calcium to the basal level, which induces the release of rhoptry protein (29). *P. falciparum* reticulocyte-binding protein homologs-1 (*Pf*RH1), present in trace amounts in rhoptry neck, was found to trigger the release of calcium ions, which further initiates the cascade. The use of *Pf*RH1 antibody inhibited the invasion process by blocking the calcium signaling and halting the interaction of EBA175 with host receptors. Therefore, it is assumed that an alternative pathway, namely, K^+^ ion-dependent pathway for the release of protein from apical organs, might exist. In spite of the above findings, the mechanism of triggering of signals to release calcium ions is yet to be probed (87). A study indicated the role of *Pf*CDPK1 in the discharge of microneme protein for invasion. The mutational analysis further analyzed the inhibition of invasion process upon mutating its active site residues (88, 89). Microneme secretion and thereby invasion was also found to be impaired on deletion of another calcium lipid-binding Doc 2 protein, *Pf*Doc2. However, the actual mechanism of action is unclear (47, 90).

### Calcium-Dependent Cell Cycle Regulation

The cell cycle of parasite is known to be regulated by the coordinated release of calcium ions. The efflux of calcium ions from ER occurs through inositol 1,4,5-trisphosphate (IP3) channels. It was demonstrated that exogenous addition of IP3 also causes the release of ions. Melatonin hormone was found to induce the production of IP3 through PLC, which further opens the Ca^2+^ channels in the ER (91–93). An increase in calcium level on incubation with tumor necrosis factor (TNF)-α caused the downregulation of *P. falciparum’s* proliferating cell nuclear antigen-1 and ultimately the retardation of parasite growth (94). Calcium mediates its activity through *Pf*CDPK7. The role of *Pf*CDPK7 was confirmed by knockout studies in which *Pf*CDPK7 mutant showed drastic retardation in growth. It can be assumed that the role of calcium might be correlated with protein kinase, *Pf*PK7 and cdc2-related protein kinase, because the inhibition of these genes also demonstrated to retard parasitic growth, but this hypothesis needs further validation (95). Expression of *Pf*CDK2 was also indicated to be at peak during the ring and trophozoite stage. However, the function of *Pf*CDK2 has not been deciphered yet (96). A different group of Ca^2+^-binding orthologs of cytoskeletal-binding protein centrin (*Pf*CEN), expressed during asexual and the gametocyte stage of parasite, was found to be colocalized with the centrosome. The role of centrin in cell division of *Plasmodium* yet concealed, while the knockout studies in *Leishmania donovani* revealed their involvement in the growth and cytokinesis (97).

There is an extensive involvement of calcium signaling in various important pathways of parasite. Any interruption would be deleterious for invasion, egress, and ultimately the growth of parasite. On these grounds, components of calcium signaling are considered for therapeutic interventions.

## Toll-Like Receptor (TLR)-Mediated Signals During Malaria

**Figure 2:**
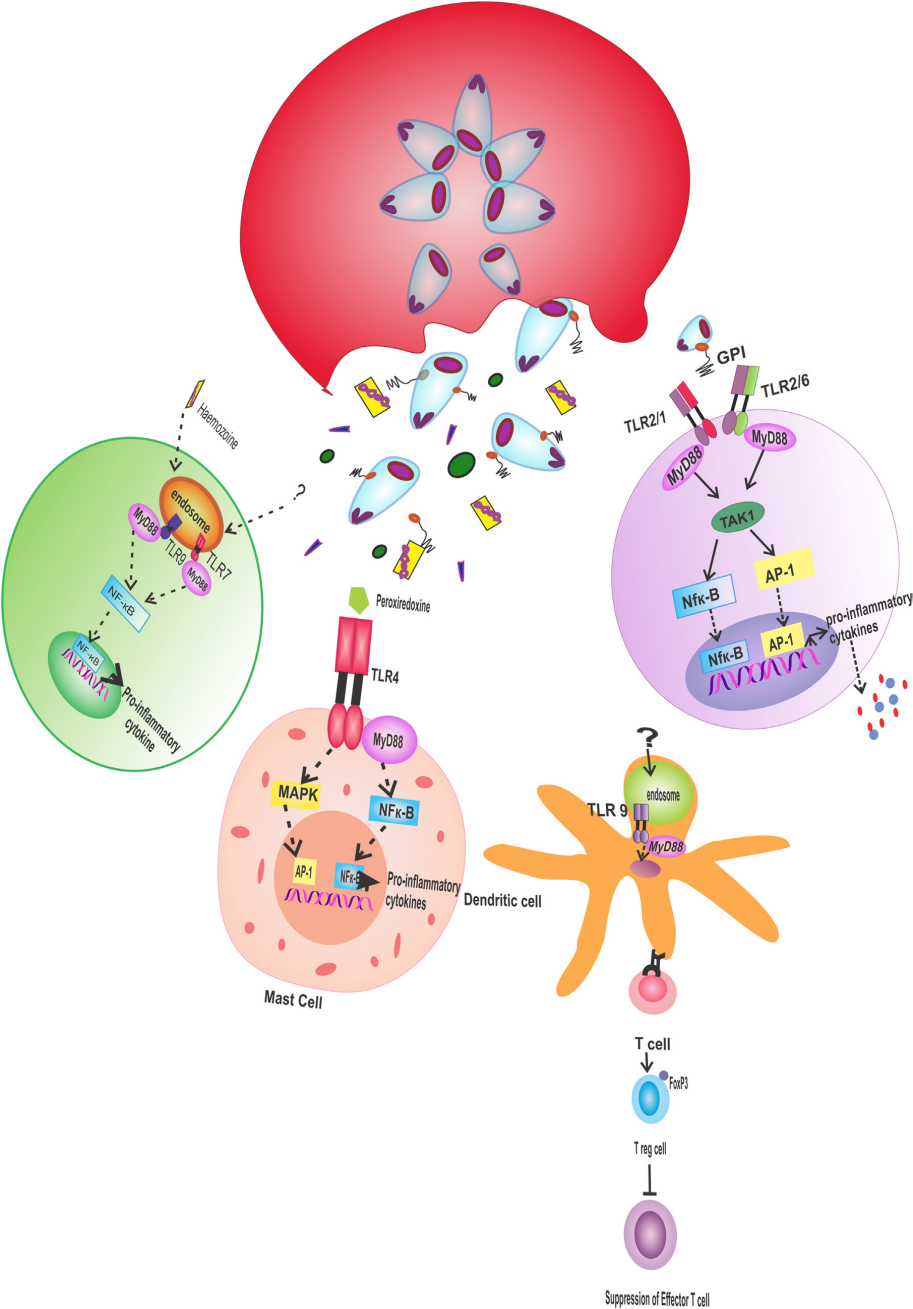
**Pro-inflammatory response through TLR: various antigenic molecules are recognized differentially by TLRs, which produce pro-inflammatory responses**. Hemozoin, released on rupture of infected erythrocyte, triggers MyD88-mediated signals through TLR9 receptor in host immune cells, and TLR7 is activated by unknown ligand to produce the same response. In dendritic cells, TLR9 activated by unknown ligand causes the differentiation of T cells to regulatory T cell, which suppress the activity of effector T cells. Antigenic protein peroxiredoxin stimulates TLR4 on mast cells, which activate MAPK and NF-κB pathway in MyD88-dependent manner, thereby ultimately releasing pro-inflammatory cytokines. TLR2 coupled with TLR 1 or TLR6 in host immune cells recognizes GPIs on parasitic surface to induce the production of pro-inflammatory cytokine through the activation of NF-κB and AP-1. TLR, toll-like receptor; MyD 88, myeloid differentiated factor 88; NF-κB, nuclear factor kappa-light-chain-enhancer of activated B cells; AP-1, activator protein 1; MyD88, myeloid differentiation factor 88; GPI, glycosylphosphatidylinositol.

Pathological symptoms during the severity of malaria are corresponding to the elevated level of pro-inflammatory cytokine. With the release of liver schizonts, a relative increase in the production of pro-inflammatory cytokine, such as interleukin (IL)-12, IL-8, and interferon (IFN)-γ, has been noticed in infected individuals (98–103). Any flaw in the inflammatory response will be responsible for the severity of disease (101, 104, 105). Antigen recognition by TLRs is one of the common mechanisms for the activation of the innate immune response. The mechanism involves the triggering of the signals from TLRs, which will ultimately cause the activation of the pro-inflammatory response (106–108) (Figure 2). In a case study of severe malaria, higher levels of TLR2, TLR4, and TLR8 were found (109). Owing to their role in severe malaria pathologies, TLRs are considered to be good candidate for in-depth research to elucidate the mechanism of innate immune response during parasite infection. Also, TLR ligands can be implicated for therapeutic intervention (110). The TLR role was investigated by correlating liver inflammation with parasite infection. In this study, cytotoxic activity of hepatic lymphocytes was induced by IL-12, produced in response to TLR-myeloid differentiation factor 88 (MyD88) mediated signaling pathway. On the contrary, the normal IL-12 level was found in MyD88-deficient mice. The cascade initiates with the interaction of a ligand with TLR extracellular domain, which is followed by the transfer of signal to intracellular Toll/interleukin-1 receptor (TIR) domain (111). Afterward, the signals are transferred to downstream targets and followed by the activation of transcription factors like nuclear factor kappa-light-chain-enhancer of activated B cells (NF-κB) and activator protein 1. The signals from the TIR domain are transduced *via* intracellular adaptors such as MyD88, TLR2, TLR4, TLR9, and TLR7 (112).

### Role of TLR9

During malaria, TLR9 is involved in providing protective immune response in a MyD88-dependent manner (113). Hemozoin (HZ) was indicated to stimulate this response (114–116). In a study, the ligand property of purified HZ against TLR was studied. The studies performed in TLR9-deficient mice showed the inadequate functioning of HZ in the production of chemokine, cytokine, and costimulatory signals (117). Parroche et al. (118) identified that natural HZ but not purified hemin can induce TLR9. Experimentally, it was explored that the stimulation was forbidden due to the lack of binding to TLR9 in the presence of nuclease. Later, it was confirmed that DNA present on the surface of HZ interacts with TLR9 (118). Another piece of information supporting the role of TLR signaling during malaria was derived from the finding, which has demonstrated the activation of regulatory T (Treg) cells in a TLR9-dependent manner. During the *P. yoelii* infection, an unknown protein of parasite stimulates the TLR9 present on dendritic cells. These signals ultimately trigger off the Treg cells, which in turn suppress the effector T cells. Evidence suggests that mice deficient in TLR9 are more resistant to malaria infection due to the activation of the effective response of T cells (15).

### Role of TLR4

The pro-inflammatory response is not restricted to be mediated through TLR9, but in recent past, natural HZ has been shown to bind with host fibrinogen, which interacts with fibrinogen receptor TLR4 on monocytes, and this interaction leads to downstream activation of NF-κB and MAPK, thereby arousing the oxidative burst and elevated expression of TNF and other pro-inflammatory cytokines (119). Similarly in case of murine malaria, 2-Cys peroxiredoxin antigenic protein of *Plasmodium* stimulates TLR4 on mast cell and macrophages to produce TNF-α (120) (Figure 2).

### Role of TLR7

A study carried out in *Plasmodium chabaudi* elucidated the role of TLR7 in the production of IFN-1, IL-12, and IFN-γ. Earlier, TLR9 was reported as the key sensor of infection. But the experiments conducted in the absence of TLR7 and MyD88 showed the remarkable reduction in the pro-inflammatory cytokine-like IFN-1. Contrary to it, no influence on IFN production was observed in mice deficient in TLR2, TLR4, TLR9, interleukin-1 receptor, or IL18R. Rational for this disparity is that the activation of TLR9 or TLR 7 depending on the time or stage of infection and alteration in the available ligand (16). Although the parasitic ligand triggering TLR7 has not yet been proven, however, based on finding that single-stranded RNA is required for TLR7-dependent production of IFN-I during viral infection (121), it was hypothesized that the RNA of parasite might work as a ligand against the receptor (16). Despite this speculation, the actual receptor–ligand interaction needs to be elucidated.

### Role of TLR2

TLR2 recognize glycosylphosphatidylinositol (GPI) with TLR1 and TLR6 in a heterodimeric form (110) and induces the inflammatory cytokine production (122) (Figure 2). Severity of malaria was found to be correlated to allelic variation in TLR1 (123). TLR2 signaling in liver stage initiates the production of pro-inflammatory response, which hampers the parasite’s development (124).

## GPI-Based Signaling and Immune Response

Several factors are involved in immune modulation leading to malaria pathologies. For instance, GPI is involved in the elicitation of innate immune response. GPI, ubiquitously found in eukaryotes, but are more prominent on parasitic surface. GPIs of different species exhibit structural diversity. GPIs are considered as toxic due to their deleterious effect of inducing of pro-inflammatory cytokines such as TNF-α, IL-1, IL-6, IFN-γ, and nitric oxide (NO) in macrophage. The induced cytokines lead to the development of symptoms like hypoglycemia, pyrexia, fever, illness, and lethal cachexia (125–129). Conversely, the anti-GPI antibody significantly diminishes the pro-inflammatory response. Consistent with this, data show that surviving individuals after sever malaria have higher level of anti-GPI (130, 131). The GPI manifest its effect through the activation of protein tyrosine kinase and phosphokinase C consecutively activating NF-κB (125, 129, 132). Studies with knockout mice demonstrated that TLR-2 (110) and to a lesser extent TLR-4 (133) recognize GPIs, on the surface of merozoites (Figure 2). Mice deficient in MyD88 and CD36 showed reduced TNFα secretion in the presence of GPI. This indicates that GPI pass down its signals through TLR2 and CD36 (134). Elucidating the signaling cascade activated by GPI in murine peritoneal and bone marrow-derived macrophages, it was found that GPIs from *Plasmodium* can differentially stimulate the MAPK pathway like ERK, P38, and c jun *N*-terminal kinase (JNK) (128, 134). Of the above three MAPK pathway, ERK is not involved in GPI-induced secretion of TNF-α and NO (128). Reflecting on JNK its two isoforms, JNK1 and JNK 2 participate differentially in GPI-mediated cytokine production. On the induction of macrophage with the GPI, unaffected production of IL-6 and NO was observed in both JNK1^−^/^−^ and JNK2^−^/^−^. But IL-12 and TNF-α levels were reduced in JNK2^−^/^−^, thus indicating the essential requirement of JNK2 to produce TNF-α and IL-12 (134). The crucial role of GPI in the activation of pro-inflammatory response and highly conserved nature suggest the synthetic GPIs as potential vaccine candidates (135). Targeting GPIs for designing the antimalarial therapy would be beneficial and secured from emergence of drug resistance (136). GPIs exerts their effect by imitating host GPIs, thereby modulating the normal host signaling pathways, thus candidature of GPI as a vaccine target was further verified (136, 137).

In an effort to find inhibitory molecules against GPIs a molecule, human C1 inhibitor (C1INH) with an anti-inflammatory effect, secreted by liver cell, was brought to attention (138). C1INH directly interact with GPIs of *P. falciparum*, and this binding inhibits the invasion of parasite to fresh erythrocyte and also it obstructs the interaction of iRBC to CD36 and chondroitin sulfate A, thereby halting parasite sequestration and consequently suppress the production of the pro-inflammatory cytokine as well (139). The effectiveness of C1INH was questioned due to the insufficiency of the endogenously produced molecule in controlling the disease pathogenesis. The presence of a mechanism like elastase, responsible for the weakening of the effect of C1INH in *Pseudomonas aeruginosa*, was hypothesized to be present in *P. falciparum* (139, 140) but has no experimental clues.

## Cytoadhesion and Related Signaling During Malaria Infection

Most of the malaria pathologies are associated with the cellular interaction between host and parasitic proteins (7, 10, 141) (Table 1). Therefore, the focus on the response produced by cytoadhesion will help in overviewing of mechanisms of pathogenesis. During trophozoite stage, a large repertoire of proteins is exported on iRBC surface. Of hitherto of proteins, interaction of *Pf*EMP1 with endothelial cell surface receptors or receptors on immune cells is most widely explored (142–145). Most of the exported proteins form a knob-like structure with adhesive property (4). Binding of these proteins to the EC, other iRBC or fresh RBCs cause the sequestration of iRBC in different tissue. As a consequence, it reduces the blood flow and bypasses the main flow to stay off from the splenic clearance (146). On the EC, several different types of receptors such as CD36, interstitial cell adhesion molecule (ICAM-1), endothelial protein C receptor (EPCR), P-selectin, and E-selectin are expressed in a tissue-specific manner (10, 141, 147, 148).

Recently, it was suggested that shedding of protective glycocalyx from endothelium is responsible for increased permeability and procoagulation state (149, 150). It makes direct access of the iRBC on the endothelial receptors. This finding provides a new line for the development of adjunct therapies, which can prevent the damage to the glycocalyx (151).

CD36 scavenger receptor is an important signaling molecule for various ligands and responsible for producing a pro-inflammatory response during inflammatory disease. The importance of CD36-mediated sequestration in parasitic growth was demonstrated through mutant of *P. berghie* with deficient CD36-binding ligand (152). The adherence of the iRBC to CD36 on vascular endothelium activates the intracellular signaling cascade, which in turn intensifies the affinity of the interaction of receptor for its ligand (11, 153). A downstream signaling cascade of CD36-iRBC interaction was explained by employing cross linking anti-CD36 or recombinant domain CD36-binding domain of *Pf*EMP. This interaction stimulates src-dependent kinase, which activates ecto-ALP present on the surface of the EC (Table 1). The ALP subsequently potentiates the affinity of the receptor by dephosphorylation (153). It has been shown that recruitment of α_5_β_1_ integrin due to src signaling is responsible for an increase in affinity. This also leads to the rearrangement of cytoskeleton protein through phosphorylation of (P130Cas) Crk-associated substrate (P130Cas) adaptor protein (61, 154). The src kinase also activates Erk1/2, but it is not involved in receptor interaction. In monocytes, it avails in phagocytosis of iRBC but not concerned with TNF, responsible for sever pathologies (155, 156). During acute lung injury in *P. berghei* infection, splenic monocytes recruited to the lung tissue cause CD36-mediated phagocytosis of iRBC (157). Future research is expected for unrevealing the prospects of phagocytosis and the involvement of this pathway in activation of adaptive immunity.

In childhood malaria, *Pf*EMP1 containing domain cassettes 8 and 13 domain binds to specific EPCR. EPCR-bound activated protein C (APC) activates protease activation receptor-1, which leads to induction of protective signals. APC on getting released binds to membrane phospholipids on platelets and causes the inactivation of the coagulation factor (14). However, during malaria, the interaction of *Pf*EMP1 with EPCR inhibits the activation of protein C then pro-inflammatory cytokines from EC cause the shedding of EPCR (158). But it is matter to ponder upon that whether to implicate EPCR-mediated interaction for restricting iRBC sequestration? Would providing APC exogenously restore the cytoprotective and anticoagulant state? It would then provide novel concept to deal with the severity of malaria (158).

Host immune response causes the subtle changes in the functioning of blood–brain barrier, which affects the cellular trafficking of lymphocytes (159). The infiltration of lymphocytes from endothelium is mediated through the interactions between ICAM-1 and cell surface integrin (160). In *P. falciparum* infection, interaction of iRBCs with ICAM-1 is one of the reasons for the development of cerebral pathologies (161, 162). During the inflammatory response, the expression level of ICAM-1 increases. The rosetting of iRBC and their adherence to ICAM-1 was suggested to be responsible for bacterial enteric infection in children with severe malaria (163). Severity of disease involves damage in the microvasculature and organ. So, implementation of anti-adhesion agents coupled with anti-malarials are needed to combat the disease (164). The application of antiadhesion therapies would provide a new perspective in the reduction of the interactions related to severe pathologies. In the line of this effort, a truncated ICAM-1 biophore peptide (IBT213) was designed through *in silico* approach, which can specifically block the binding of *Pf*EMP1 to ICAM-1(165).

## Conclusion

The impression of the involvement of signaling pathways during the asexual stage of parasite is clear. The different signaling strategies are involved to gain access or to modulate the host environment. Several techniques such as pharmacological inhibition and reverse genetic approach and techniques for single-cell live imaging revealed various indispensable components responsible for the invasion, egress, parasite growth, and survival. The non-homologous nature of most of the signaling molecules such as *Pf*PKG, *Pf*PKA, and *Pf*CDK gives an opportunity to exploit them as targets. The uniqueness of these molecules to the Apicomplexan provides an additional benefit. Despite this, several host receptors such as TLRs and immune cells are also awaiting (112) for a ligand search. Along with the ligand screening, a few parasitic molecules involved in the regulation of parasite signals are remained to be explored. More emphasized and detailed studies focused on the characterization of such signaling molecules will provide a new insight for inviting valuable treatment therapy to combat malaria.

## Author Contributions

RS, DS, PR, and BS collected the data. RS wrote the manuscript. Overall monitoring was done by TB.

## Conflict of Interest Statement

The authors declare that the research was conducted in the absence of any commercial or financial relationships that could be construed as a potential conflict of interest.
